# Listening Effort During Sentence Processing Is Increased for Non-native Listeners: A Pupillometry Study

**DOI:** 10.3389/fnins.2018.00152

**Published:** 2018-03-13

**Authors:** Giulia Borghini, Valerie Hazan

**Affiliations:** Department of Speech Hearing and Phonetic Sciences, Faculty of Brain Sciences, University College London, London, United Kingdom

**Keywords:** non-native speech perception, pupillometry, listening effort, speech perception in noise, cognitive load

## Abstract

Current evidence demonstrates that even though some non-native listeners can achieve native-like performance for speech perception tasks in quiet, the presence of a background noise is much more detrimental to speech intelligibility for non-native compared to native listeners. Even when performance is equated across groups, it is likely that greater listening effort is required for non-native listeners. Importantly, the added listening effort might result in increased fatigue and a reduced ability to successfully perform multiple tasks simultaneously. Task-evoked pupil responses have been demonstrated to be a reliable measure of cognitive effort and can be useful in clarifying those aspects. In this study we compared the pupil response for 23 native English speakers and 27 Italian speakers of English as a second language. Speech intelligibility was tested for sentences presented in quiet and in background noise at two performance levels that were matched across groups. Signal-to-noise levels corresponding to these sentence intelligibility levels were pre-determined using an adaptive intelligibility task. Pupil response was significantly greater in non-native compared to native participants across both intelligibility levels. Therefore, for a given intelligibility level, a greater listening effort is required when listening in a second language in order to understand speech in noise. Results also confirmed that pupil response is sensitive to speech intelligibility during language comprehension, in line with previous research. However, contrary to our predictions, pupil response was not differentially modulated by intelligibility levels for native and non-native listeners. The present study corroborates that pupillometry can be deemed as a valid measure to be used in speech perception investigation, because it is sensitive to differences both across participants, such as listener type, and across conditions, such as variations in the level of speech intelligibility. Importantly, pupillometry offers us the possibility to uncover differences in listening effort even when those do not emerge in the performance level of individuals.

## Introduction

Due to increased mobility, a growing number of people work or study on a daily basis in a second language environment. Challenges for non-native listeners arise both because their knowledge of the language is imperfect, and because they are more affected by adverse listening conditions, such as the presence of background noise or other interference. These difficulties occur for beginner learners, but also persist after years of exposure, even after speakers and listeners have gained experience, practice, and confidence in the non-native language. However, little is known about the underlying cognitive effort required to understand speech in a second language. In the present research, we used pupillometry to investigate differences in listening effort in native and non-native listeners during speech perception at matched intelligibility levels.

It is well-known that the detrimental effect of noise and of environmental signal distortion on speech perception is much stronger when listening in a second language (L2) rather than in one's native language (L1). Highly competent non-native listeners are significantly less accurate than native listeners at speech recognition in the presence of noise or reverberation, even when performance was native-like under favourable listening conditions (Takata and Nábělek, 1990; Mayo et al., 1997; Cutler et al., 2008). Indeed, speech perception abilities are shaped and modulated by linguistic experience in order to maximise the sensitivity to those acoustic contrasts that are important to discriminate meaning in the specific linguistic community the individual belongs to. Non-native listeners may use different or fewer acoustic cues for phoneme discrimination compared to native listeners. For example, Japanese adults, when required to discriminate the English phonemes /r/ and /l/ are most sensitive to changes in the second rather than the third formant even though this acoustic cue is irrelevant to discriminate between these phonemes (Iverson et al., 2003). Moreover, it has been demonstrated that when a high degree of cognitive effort is required simultaneously with the speech recognition task, native speakers rely more on contextual plausibility than on acoustic cues, while non-native listeners do not (Mattys et al., 2010). These results specifically suggest an increased difficulty for non-native listeners in exploiting lexical information, particularly under high cognitive load, presumably due to a deficient lexical and semantic knowledge. It has also been shown that non-native listeners require a higher signal clarity (e.g., the use of a clear speaking style) in order to fruitfully access contextual cues (Bradlow and Alexander, 2007).

When investigating speech perception in non-native listeners, it is also important to take into account the contribution of working memory. Indeed, language perception and understanding is an on-line process, in which listeners have to match the incoming variable and fast speech signal against representations of words stored in memory. In order to be able to efficiently understand a conversation, multiple potential interpretations of the incoming signal need to be evaluated in real time as soon as a portion of the stream is available for the listener (Garcia Lecumberri et al., 2010). Listeners have to hold in working memory the audio signal while comparing it to potential lexical alternatives retrieved from the lexicon, and also temporarily hold these alternatives in working memory to make them available for comparisons until the word has been disambiguated. It is therefore obvious that a good working memory capacity, which determines the ability to simultaneously store and process information (Rönnberg et al., 2013), is inherently necessary for lexical access. Working memory also has an important role for on-line language processing during conversation; it is used to maintain relevant semantic information, inhibit the processing of irrelevant stimuli, and for selectively attending to a specific audio stream.

In the literature, the Ease of Language Understanding (ELU) model (Rönnberg et al., 2013) indeed stresses the on-line feature of speech processing, with the retrieval of lexical representations stored in long term memory playing a central role during the word recognition process. Further, it claims that additional explicit working memory is required whether there is a mismatch between the speech signal input and the long term memory representation it is compared with. When communication takes place in ideal listening conditions, the linguistic input rapidly and automatically matches the mental lexical representation with a high enough degree of precision, and lexical access proceeds quickly and without additional explicit effort. However, when communication happens in sub-optimal listening conditions (i.e., due to signal distortion, background noise, non-native listener/speaker), an explicit contribution of working memory is necessary to support listening and to resolve the mismatch occurred, leading to an increased cognitive effort. This additional explicit processing loop helps fill in missing information, using both phonological and semantic knowledge stored in long term memory. According to the ELU model, explicit and implicit processes run in parallel, the former being rapid and automatic, the latter being slower and more demanding in terms of cognitive resources, and together modulating the working memory demand during speech perception.

If we consider the on-line process of non-native speech perception in the light of the ELU model, we can identify several steps in the recognition process in which working memory demand is increased relative to first language comprehension. First, the availability of candidate words and their online selection strictly depends on accurate phoneme perception and representation. Word candidates might be erroneously activated if the listener's impoverished L2 phonemic perception fails to rule them out, and this can lead to a delayed resolution of word competition for non-native compared to native listeners (Cutler et al., 2006). In addition, the lexical knowledge of a listener in their L2 may be extremely reduced relative to that of their first language so the target word may not even be available for selection. Interestingly however, a previous study reported a remarkably similar effect of background noise on native and non-native listening, when a set of candidate words from the target language was considered (Scharenborg et al., 2017). The presence of noise resulted in an increased number of candidate words considered for recognition in both listeners' groups. Nevertheless, the study did not consider the possibility of additional activation of words in the listener's first language during non-native speech perception. Indeed, the L2 listener's competitor set may also contain words from the lexicon of their native language, which would make the selection process more effortful. It has been demonstrated by studies using eye-tracking techniques that even experienced non-native listeners during a word recognition task often activate words from their first language in parallel with words from the language they are attending to Spivey and Marian (1999). This added competition has also been shown to be hard to overcome for L2 learners (Broersma and Cutler, 2011). Finally, as discussed above, we know that higher-level processes, such as relying on semantic context, help resolve lower-level (perceptual) ambiguity that can arise both from a poor phoneme representation, and from any kind of signal distortion or degradation. However, L2 listeners' experience of syntax, and their contextual and pragmatic knowledge are limited, and therefore less effective in resolving phonological or lexical ambiguity. So, while it would be helpful for L2 listeners to rely more heavily on higher-level context to compensate for poorer perceptual abilities, their-higher level resources are less effective than in native listeners.

In situations in which L2 listeners are able to perform at native-like levels via an increase in cognitive effort, differences in listening difficulty will not become apparent if only behavioural performance is examined (Zekveld et al., 2010). In real life however, this additional cognitive cost is likely to entail an increased fatigue and a reduced ability to multi-task. This awareness of the limitation of only considering behavioural performance in speech understanding combined with awareness of the role of cognitive processing in speech perception (Akeroyd, 2008; Besser et al., 2013) has led to an increased interest in the study of listening effort during speech processing.

Pupillometry, the measurement of task-related pupil dilations, has been used in language research for around 50 years. Pupil responses have been shown to be sensitive to intelligibility levels (Zekveld et al., 2010), degree of spectral degradation of the signal (Winn et al., 2015), masking condition (Koelewijn et al., 2012a; Zekveld et al., 2014), syntactic complexity, and sentence length (Piquado et al., 2010). Moreover, higher cognitive abilities such as working memory capacity and linguistic closure ability have been shown to correlate with a greater pupil response and a longer peak latency of pupil dilation (Zekveld et al., 2011; Koelewijn et al., 2012b). Crucially, previous research has established that the cognitive processing load evoked by speech perception can be dissociated from actual speech perception performance, i.e., the amount of information correctly understood. For example, using pupil dilation measures, studies have reported a variation in the level of listening effort even at matching levels of intelligibility. Those variations were associated with the use of different kinds of masking noise and changing levels of task demand, but not with variations in speech perception performance (Mackersie and Cones, 2011; Koelewijn et al., 2012a). However, despite the large body of research in the field of non-native language perception, and the increased interest in measuring listening effort, few studies have used pupillometry to investigate non-native speech comprehension. A study considering the complex task of simultaneous translation, proved among other results that repeating back words in a non-native language entailed an increased pupil dilation compared to the same task performed in the speaker's native language (Hyönä et al., 1995). More recently, a pupillometry study investigating spoken word recognition considered the performance of three groups of participants: monolingual English speakers, early and late Spanish-English bilinguals (Schmidtke, 2014). Pupil response was delayed for bilingual compared to monolingual listeners, and a larger neighbourhood effect was obtained for bilingual compared to monolingual listeners. Researchers also reported a greater word frequency effect for late bilingual compared to monolingual and early bilingual individuals, with an increased mental effort required to retrieve less common words. Interestingly, within bilingual participants, higher English proficiency was associated with an earlier pupil response, and with a smaller effect of word frequency and neighbourhood density. However, this previous study only considered single word recognition in quiet, without therefore directly addressing the challenges of everyday communication. Another study combining eye-tracking and pupillometry investigated the added cognitive load needed for bilingual individuals to process language switches within a sentence. It was showed that bilinguals, both at the beginning of development and in adulthood, are affected by language switches in terms of increased cognitive load, even when listening to simple sentences (Byers-Heinlein et al., 2017). Although providing interesting insights on the mind's ability to cope with a complex language environments, this study does not address the additional challenges faced by non-native listeners who acquired a second language later in life, and often need to deal with suboptimal listening conditions.

The purpose of the present study is to gain insights into the factors affecting listening effort in non-native listeners, by comparing native and non-native listeners' pupil response during a speech perception in noise task. Specifically, we compared the listening effort experienced by native and non-native participants when their performance in the speech perception task is matched. The primary aim of this experiment is to compare the listening effort for native and non-native listeners at two matched levels of speech intelligibility in order to investigate: (i) whether native and non-native listeners performing at the same accuracy level differ in terms of cognitive effort required, (ii) whether intelligibility level differentially modulates the listening effort for native and non-native participants (e.g., if the same increase in task difficulty leads to a greater increase in listening effort for non-native individuals). To our knowledge, no previous study has applied pupillometry to investigate differences in listening effort between native and non-native listeners during a sentence processing task in noise, at equated levels of intelligibility.

We predicted that the listening effort reflected by the pupil response would be higher for non-native listeners when compared to native listeners for a given intelligibility level. This is because we expected listeners to allocate a greater amount of cognitive resources when attending to a second language compared to their native language. We also hypothesised that increases in task difficulty would cause pupil response to change at a steeper rate for non-native compared to native listeners, because of the previously documented increased detrimental effect of noise on non-native compared to native speech perception. Additionally, we expected that the listening effort reflected by the pupil response would be higher when the intelligibility level is lower compared to when it is higher, in line with previous research.

## Materials and methods

### Participants

Fifty adults from two different language backgrounds took part in the experiment. The first group included 23 native British English participants (15 women and 8 men), aged 18–32 years (*M* = 23.3, *SD* = 4.2 years). The second group included 27 participants (18 women and 9 men) with Italian as L1 and English as L2, aged 20–35 years (*M* = 28.4, *SD* = 4.1). All participants had been living in the UK for at least 10 months. Participants were recruited from the UCL Psychology subject pool and from social media. They reported not to suffer from cataracts or diabetes, and to not have used drugs or medications in the 48 h prior the experiment. Moreover, they were able to fixate the cross appearing on the screen without glasses or contact lenses. These selection criteria were chosen because of their potential impact on pupil dilation. All participants provided written informed consent to participate and received a monetary compensation for their participation. The study was approved by the Ethics Committee at University College London.

### Stimuli and tests

#### Background tests

All participants were screened using pure tone audiometry to ensure that their hearing thresholds were 20 dB HL or better at octave frequencies between 250 and 8,000 Hz. At the beginning of the experimental session, all participants carried out a set of background tests. The aim of these tests was to obtain a cognitive profile for each participant including measures which previous research suggested to be related with the ability to perform a speech perception task in noise (Flege et al., 1999; Besser et al., 2013). Specifically, for each participant the following tests were administered:

- Digit span, forward, and backward (Wechsler et al., 2008). This is commonly used as a measure of verbal working memory storage capacity. The test was administered in the participant's first language (either English or Italian).
- Phonological short term memory test: the Children's Test of Non-word Repetition (CN-Rep) (Gathercole et al., 1994). This consists of 40 non-words from 2 to 5 syllables length (e.g., “diller,” “defermication”) preceded by 2 practice items. Answers were recorded and evaluated *post-hoc*.

In addition, non-native participants were asked to complete an on-line linguistic background questionnaire designed to collect information about their level of self-reported English proficiency, their language usage, and their perceived cultural identity. The questionnaire was designed by adapting questions from two different sources: the Language History questionnaire (Li et al., 2014) and the Language Experience and Proficiency Questionnaire (Marian et al., 2007). Participants were also recorded while reading aloud a short story, “Arthur the rat” (MacMahon, 1991). A British English native speaker (without TEFL training) not involved in the study subsequently rated the degree of foreign accent of their speech on a scale from 1 (= native-like) to 7 based on a sentence extracted from the speech recorded. Given that all non-native participants were from the same L1 background (Italian) and that the same sentence was used for the rating, the rating provided us with a measure of relative accent within the L2 participant group. The aim of these tests was to obtain an accurate linguistic profile for the non-native participants included in this study, in order to later be able to explore any correlation between listening effort and language use and proficiency.

#### Experimental stimuli

Sentences presented in the study were taken from the Basic English Lexicon (BEL) sentence materials (Calandruccio and Smiljanic, 2012) which include 20 lists of 25 sentences. BEL sentences were specifically developed to test speech recognition for various listener populations, therefore they contain lexical items and syntactic structures appropriate for use with non-native listeners. Each sentence has four keywords, which were used to score comprehension. Examples of the sentences are: “*The PARK OPENS in ELEVEN MONTHS*,” “*My DOCTOR WORKS in that BUSY HOSPITAL*” (key words in capital letters). Sentences were recorded in an anechoic chamber and produced by four native British English speakers (two females) at a natural self-paced rate. Sentence duration was between 1.6 and 2.6 s. Recordings were root-mean-square (RMS) normalised to an average amplitude of 65 dB. Overall, each participant was presented with 8 experimental blocks of 15 trials each (120 sentences in total). For each experimental block, a list was randomly selected. From the selected list, only 15 sentences per block were randomly chosen and presented to the participant. Each sentence was only played once during the entire experimental session for a given participant.

#### Experimental task

The experimental task was a speech intelligibility test: participants were asked to listen to sentences and repeat them back to the experimenter. A loudspeaker was used for the presentation of auditory stimuli in order to ensure the participants' comfort and avoid pupil measurement being affected by discomfort that could be caused by wearing headphones. The experimental task consisted of three speech perception tests: a first one performed in quiet, and the remaining two performed in noise, with speech masked by 8-talker babble noise. The main purpose of the test in quiet was to obtain a measure of intelligibility for each participant. The test in quiet was always presented at the beginning of the experimental session. This is because we wanted the measure of speech perception in quiet not to be affected by any learning effect due to previous exposure to the speech perception task in noise, particularly for non-native listeners. The presentation order of the two conditions in noise was randomised: 24 participants were presented with the high intelligibility condition first, 26 with the low intelligibility condition first. Therefore, the order of presentation should not affect the comparison across the two conditions in noise. During the three conditions, the speech level was constant at ~67–69 dB, as measured by a sound level meter. The speaker order during the test was randomised across the sentences presented, in order to avoid habituation and to increase the task's ecological validity.

##### Speech perception in quiet

Participants were presented with five practice items followed by two blocks of 15 sentences each. All the stimuli were presented in quiet.

##### Speech perception in babble background noise

For each condition, three experimental blocks were presented. For the first block, an adaptive procedure was used to estimate the signal-to-noise (SNR) level required for reaching the target intelligibility level (Levitt, 1971). Levels of 40% (“low”) and 80% (“high”) intelligibility were chosen as targets to cover a considerable range in listening effort, but without resulting in extreme conditions where perception would be either effortless or too difficult. This is because when the processing demands of a task exceed available resources, pupil responses decline, reflecting task disengagement (Granholm et al., 1996). The background noise used as a masker consisted of an 8-talker babble noise, obtained from recordings of spontaneous speech from 4 female and 4 male English native speakers. During the adaptive block, the SNR was manipulated by adapting both the speech and the masker levels so that the overall intensity level of the compound signal was fixed at 67–69 dB. The rationale for this was to avoid any confounding effects on pupil dilation of variations in overall sound intensity. The first sentence of the adaptive block was always presented at 20 dB SNR; subsequently, the SNR was manipulated to target the level at which 40 or 80% of key words were understood. The changes in step size were defined by an algorithm taking into account the participant's performance and test stage; 9 dB SNR changes were applied during the initial stage and smaller 3 dB steps subsequently. The adaptive test terminated when either there had been five reversals or 15 trials had been presented. From this adaptive procedure, the SNR values corresponding to the reversals were averaged to obtain a single SNR value. In the two following blocks, audio stimuli were presented at that fixed SNR level. The same procedure (1 adaptive + 2 fixed blocks) was repeated twice for tracking both the high and low intelligibility levels.

### Pupillometry

The pupil size and location of the left eye were measured during the speech perception tasks using an EyeLink 1000 eye-tracker. The system uses infrared video-based tracking technology, with a spatial resolution of ~0.01 mm (value calculated for a pupil diameter of 5 mm), and was positioned at a horizontal distance of 55 cm from the participant. A headrest supporting the forehead and chin of the participant was used in order to reduce movement artefacts while performing the experiment. Pupil data were collected at the sampling rate of 500 Hz, and were stored in a connected PC. During data collection, the experimenter was able to visually inspect the video recording from a monitor, and to take action if needed (e.g., reminding the participants to fixate the centre of the screen, asking them to move in order to have the pupil in the eye-tracker searching area). The experimental task and data collection were controlled using MATLAB version R2015a. Pupil diameter was recorded during the entire duration of the three experimental conditions; event messages were included in the experimental script, so that the onset and end of each trial and each audio stimulus was time locked to the pupil data.

The pupil data were pre-processed using the following steps:

Pupil diameters below three standard deviations of the mean pupil diameter for the trial were considered as blinks. Linear interpolation was performed using the 50 data points preceding and following the blink. When more than 20% of the blinks for one experimental block happened in one trial, the trial was excluded. A smoothing first-order 10 Hz low-pass filter was applied in order to reduce the high frequency noise in the data, that were then down-sampled to 50 Hz. Lastly, the pupil data were visually inspected for artefacts. After exclusions, an average of 96% of trials per participant were included. From the continuous stream of pupil diameter data points, the section starting from 2 s prior to sentence onset (which was regarded as baseline) and ending 6.8 s after sentence onset was included in the analysis. Since sentence duration was between 1.6 and 2.6 s, the time window considered for the analysis ended between 4.2 and 5.2 s after stimulus offset. The rationale for excluding any data point beyond 6.8 s from sentence onset was that these measurements were only available for a small number of sentences and therefore any average would be calculated over very limited data.

Following the pre-processing, pupil data were averaged separately for each participant per conditions: quiet, high, and low intelligibility level. Four pupil outcome measures were obtained from the average trace of each participant and condition:

- Pupil baseline: the average pupil diameter in the 2 s preceding the sentence's onset.
- Mean pupil dilation relative to baseline pupil diameter between 0 and 6.8 s after the stimuli onset.
- Peak pupil dilation, as the maximum positive deviation from the baseline during the 6.8 s following stimuli presentation.
- Latency of the peak dilation amplitude.

### Procedure

The test was administrated in a sound-attenuated booth, with the participant seated on a comfortable chair. First, the audiometric assessment and background tests were performed. For the intelligibility tests, participants placed their chin in the head stabiliser in front of a screen positioned 70 cm away. The luminance of the room was individually adjusted so that the pupil of the participant was approximately in the middle of its dynamic range, in order to prevent ceiling and floor effects, as in Zekveld et al. (2010). The illumination ranged from 65 to 110 lx. A 9-point calibration procedure was initiated and validated. Then, the experimental task was initiated and participants were instructed to maintain their gaze and focus at a fixation cross positioned in the middle of the screen, in order to maximise the accuracy of the pupil data recorded. Each trial started with the fixation cross on the participant's screen turning black, signalling participants to fixate the screen in order to properly record their baseline pupil size. After 2 s, the sentence was played, and the fixation cross remained black for 3 additional seconds following the sentence offset, in order to allow enough time for the pupil to reach its maximum dilation. For the speech in noise conditions, the babble noise started 2 s before sentence onset (corresponding to the beginning of the baseline) and ended 3 s after sentence offset, which signalled the end of the trial. After the fixation cross had turned green, participants repeated the sentence back to the experimenter who was simultaneously scoring keyword accuracy on another screen. Participants were told that they could close and rest their eyes, and move their gaze while the fixation cross was green. After the sentence was scored, the experimenter initiated the following trial, after making sure that the participant was ready to continue. A break was taken preferably at the end of each section, but pauses at any time between trials were also allowed in case participants felt tired or needed to rest their eyes.

### Statistical analyses

One way repeated-measures analyses of variance (ANOVAs), mixed design ANOVAs and *t*-tests were conducted to test whether order of tests presentation, test condition (high and low intelligibility levels) and linguistic background of participants (native or non-native listeners) affected behavioural and pupillometric data. Individual differences' effects for all participants and for non-native listeners only were investigated by computing stepwise regression analyses, in order to assess the relationship between individual performance in the background tests and behavioural and pupil response. Lastly, additional analyses using mixed effects models were performed in order to clarify the mixed results obtained from the ANOVAs and regressions. Those results are reported in the Appendix in Supplementary Material.

## Results

### Background tests

Means and standard deviations for cognitive/phonological tests and language background information are shown in Table 1. Independent-sample *t*-tests with Bonferroni correction were conducted in order to compare the performance of native and non-native listeners on the forward and backward digit span test, and the phonological short term memory test. Non-native participants performed more poorly than native participants on the forward digit span test, *t*_(41.2)_ = −3.47, *p* = 0.003. A marginally significant difference, with again lower performance for non-native participants, was also obtained for the backward digit span test, *t*_(39.7)_ = −2.43, *p* = 0.06, and for the phonological short term memory test, *t*_(48)_ = −2.55, *p* = 0.04. The two digit span tests were additionally corrected for the violation of the assumption of variances' equality.

**Table 1:** Background tests results.

**Background tests**		**Native listeners**		**Non-native listeners**	
		***M***	***SD***	***M***	***SD***
Digit span	Forward	7.5	1.4	6.3	1.1
	Backward	6.2	1.5	5.3	1.1
Short term phonological test		37.7	3	35.4	3.2
(Non-native only)	Accent rating	N/a		5.1	1.1
	Length of residence (years)	N/a		3.6	2.6
	Overall English use	N/a		50%	0.1
	Self-reported English knowledge (0–6)	N/a		4.5	0.9

### Behavioural results

Intelligibility scores in quiet are summarised in Table 2. The reported means are averaged across the two experimental blocks, excluding the practice trials, across participants. There was a significant difference in the percentage of correctly reported words in the speech in quiet task between native and non-native participants, *t*_(48)_ = −4.80, *p* < 0.001. However, the effect size for this analysis (d = 0.14) was found not to reach Cohen's convention for a small effect (Cohen, 1988). Table 3 summarises results from the speech perception task in noise, reporting intelligibility levels, averaged across the two blocks run at a fixed SNR, and the SNR levels at which the fixed procedure blocks were run. The adaptive block used to set SNR level is not included in the analysis. A mixed design ANOVA with condition (high and low intelligibility) as within-subjects factor, and language (native and non-native) as between-subjects factor showed a significant difference in performance across intelligibility levels [*F*_(1, 48)_ = 76.45, *p* < 0.001], showing a significantly higher accuracy for the high compared to low intelligibility condition, as expected. The effect size for this difference (d = 1.87) was found to exceed Cohen's convention for a large effect (Cohen, 1988). The main effect of language group and the interaction were both found not to be significant, showing therefore that intelligibility levels did not vary across the native and non-native participants, showing that the adaptive procedure was successful in achieving matched intelligibility across groups. As expected, for each intelligibility level, the SNR levels for native listeners were significantly lower than those required by non-native listeners: *t*_(48)_ = 5.95, *p* < 0.001 for the high intelligibility condition, *t*_(48)_ = 5.97, *p* < 0.001 for the low intelligibility condition.

**Table 2:** Descriptive statistics of the behavioural results for speech perception in quiet.

**Behavioural results in quiet**						
	**All participants**		**Non-native**		**Native**	
	***M***	***SD***	***M***	***SD***	***M***	***SD***
Performance (% correct)	94.7	8.5	90.2	9.6	99.9	0.3

**Table 3:** Descriptive statistics of the behavioural results for speech perception in noise.

	**Behavioural results in noise**											
	**Babble masking/high intelligibility**						**Babble masking/low intelligibility**					
	**All**		**Non-native**		**Native**		**All**		**Non-native**		**Native**	
	***M***	***SD***	***M***	***SD***	***M***	***SD***	***M***	***SD***	***M***	***SD***	***M***	***SD***
Performance (% correct)	71.3	14.2	70.3	13.4	72.5	15.3	43.8	15.2	42.1	15.6	45.7	14.9
SNR	−4.5	4.4	−1.9	3.9	−7.6	2.5	−8.8	3.7	−6.6	3.1	−11.4	2.4

It is worth noting that, although 80% intelligibility level was targeted for the high intelligibility condition, the average keyword intelligibility level was closer to 70%. This is likely to be due to a relatively small number of trials presented in the adaptive procedure block. Importantly however, as reported above, performance levels did not vary significantly across language groups for both intelligibility conditions. Although large standard deviations were obtained, reflecting within-group variability, this was the case for both the native and non-native groups.

### Pupil data

Descriptive statistics for the pupil data are reported in Table 4 (measures in quiet), Table 5 (measures in noise), and Table 6 (measures per presentation order). These include baseline pupil diameter, mean pupil dilation, and peak dilation over the baseline, and latency of the peak following stimuli onset. For the two conditions in noise, the pupil data presented and entered in the analyses are those collected during the blocks with fixed SNR.

**Table 4:** Descriptive statistics of the pupil measures in quiet.

	**Pupil data in quiet**					
	**All participants**		**Non-native**		**Native**	
	***M***	***SD***	***M***	***SD***	***M***	***SD***
Baseline, mm	5.17	0.71	5.13	0.66	5.22	0.77
Mean dilation, mm	0.20	0.18	0.30	0.17	0.08	0.10
Peak dilation, mm	0.38	0.26	0.52	0.26	0.22	0.14
Latency of peak, sec	2.64	0.79	2.49	0.29	2.82	1.11

**Table 5:** Descriptive statistics of the pupil measures in noise.

	**Pupil data in noise**											
	**Babble masking—high intelligibility**						**Babble masking—low intelligibility**					
	**All**		**Non-native**		**Native**		**All**		**Non-native**		**Native**	
	***M***	***SD***	***M***	***SD***	***M***	***SD***	***M***	***SD***	***M***	***SD***	***M***	***SD***
Baseline, mm	5.37	0.79	5.24	0.69	5.52	0.89	5.44	0.80	5.35	0.69	5.55	0.92
Mean dilation, mm	0.13	0.16	0.18	0.17	0.06	0.13	0.18	0.20	0.24	0.19	0.10	0.18
Peak dilation, mm	0.29	0.22	0.36	0.24	0.21	0.18	0.34	0.27	0.42	0.27	0.26	0.25
Latency of peak, sec	2.66	0.72	2.79	0.71	2.50	0.72	2.63	0.76	2.64	0.83	2.61	0.68

**Table 6:** Descriptive statistics of the pupil measures in noise sorted by presentation order.

	**Pupil data in noise per presentation order**											
	**First session**						**Second session**					
	**All**		**Non-native**		**Native**		**All**		**Non-native**		**Native**	
	***M***	***SD***	***M***	***SD***	***M***	***SD***	***M***	***SD***	***M***	***SD***	***M***	***SD***
Baseline, mm	5.39	0.76	5.31	0.68	5.49	0.85	5.42	0.84	5.28	0.71	5.58	0.96
Mean dilation, mm	0.18	0.18	0.23	0.16	0.11	0.17	0.13	0.18	0.19	0.20	0.06	0.13
Peak dilation, mm	0.35	0.25	0.42	0.24	0.26	0.25	0.29	0.25	0.36	0.28	0.21	0.18
Latency of peak, sec	2.69	0.73	2.68	0.84	2.70	0.60	2.60	0.75	2.75	0.71	2.42	0.76

#### In quiet comparison

The test in quiet had some specific features that contrast with the two conditions in noise. It was always presented first, the 2 s baseline was in silence (as opposite to babble noise) and because of the nature of the test itself, the performance level was not matched between language groups. For these reasons, pupil data from the condition in quiet have been analyzed separately in order to rule out potential confounding factors, and have been excluded from the subsequent analyses.

**Figure 1:**
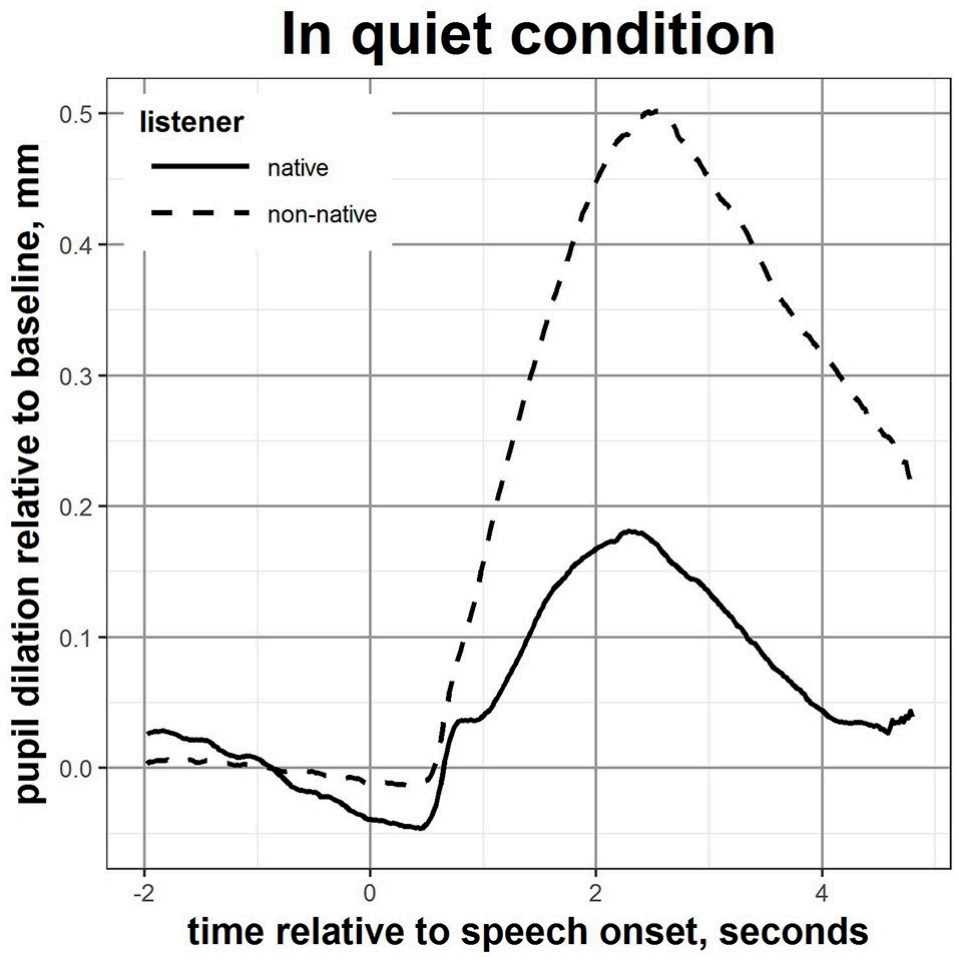
Mean pupil response over time during speech perception in quiet for native and non-native listeners.

An independent-sample *t*-test was conducted to compare the pupil response in native and non-native participants. The mean and peak pupil dilation were found to be significantly greater for non-native compared to native listeners [*t*_(48)_ = 5.52, *p* < 0.001 and *t*_(48)_ = 4.93, *p* < 0.001 respectively]. The effect sizes for these comparisons (d = 1.60 for the mean value and d = 1.43 for the peak dilation) were both found to exceed Cohen's convention for a large effect (Cohen, 1988). It is worth noting that the behavioural performance in quiet did significantly differ between native and non-native listeners, without however reaching Cohen's convention for a small effect (Cohen, 1988). Nevertheless, this yielded to a large difference in the mean and peak pupil dilation between native and non-native listeners. No statistically significant differences in the baseline and in the latency of the peak were observed between the two listeners' groups. The pupil curves dilation for native and non-native listeners during the test in quiet are displayed in Figure 1.

#### In noise comparison

**Figure 2:**
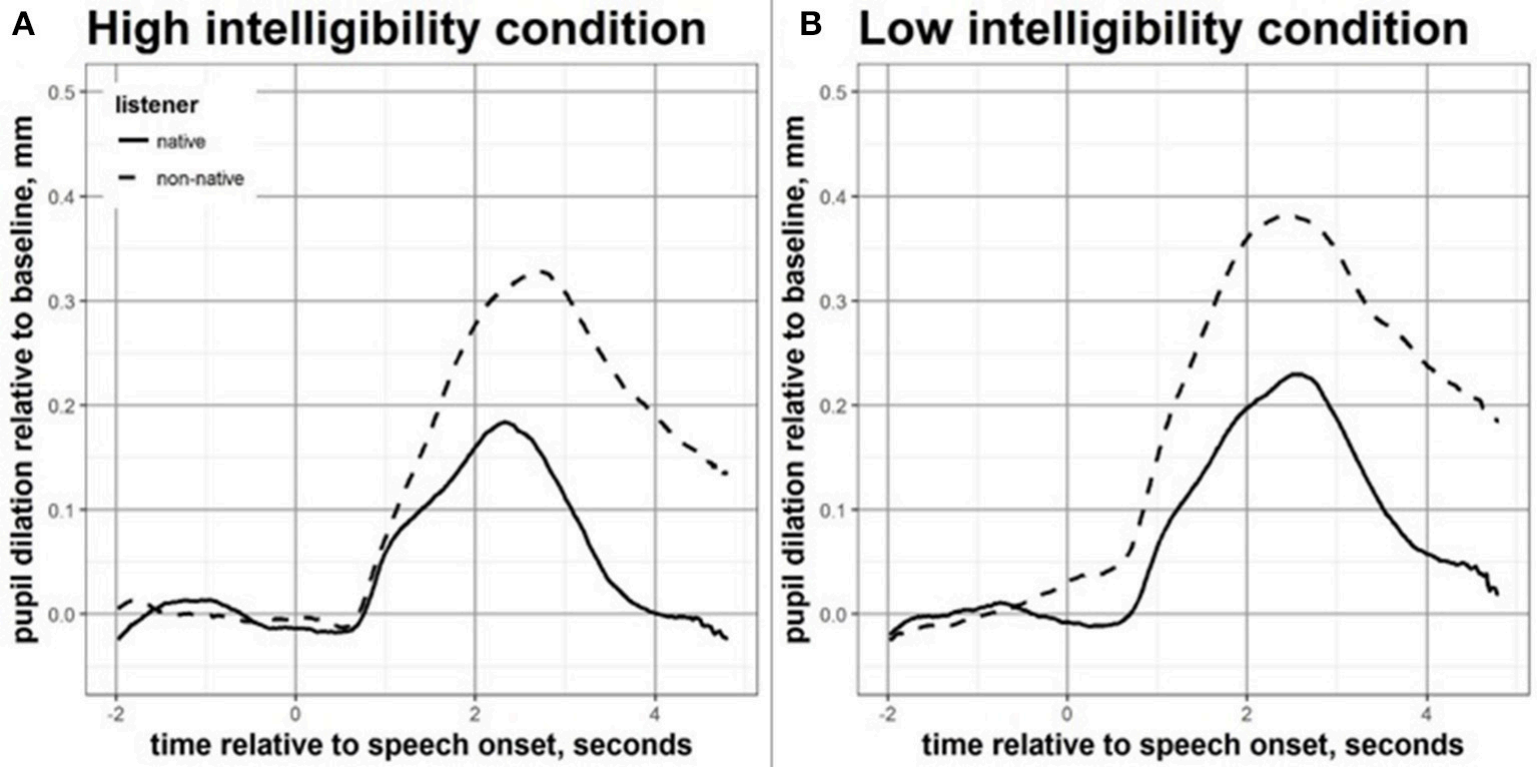
Mean pupil response over time during speech perception in noise for high **(A)** and low **(B)** intelligibility conditions, for native and non-native participants.

**Figure 3:**
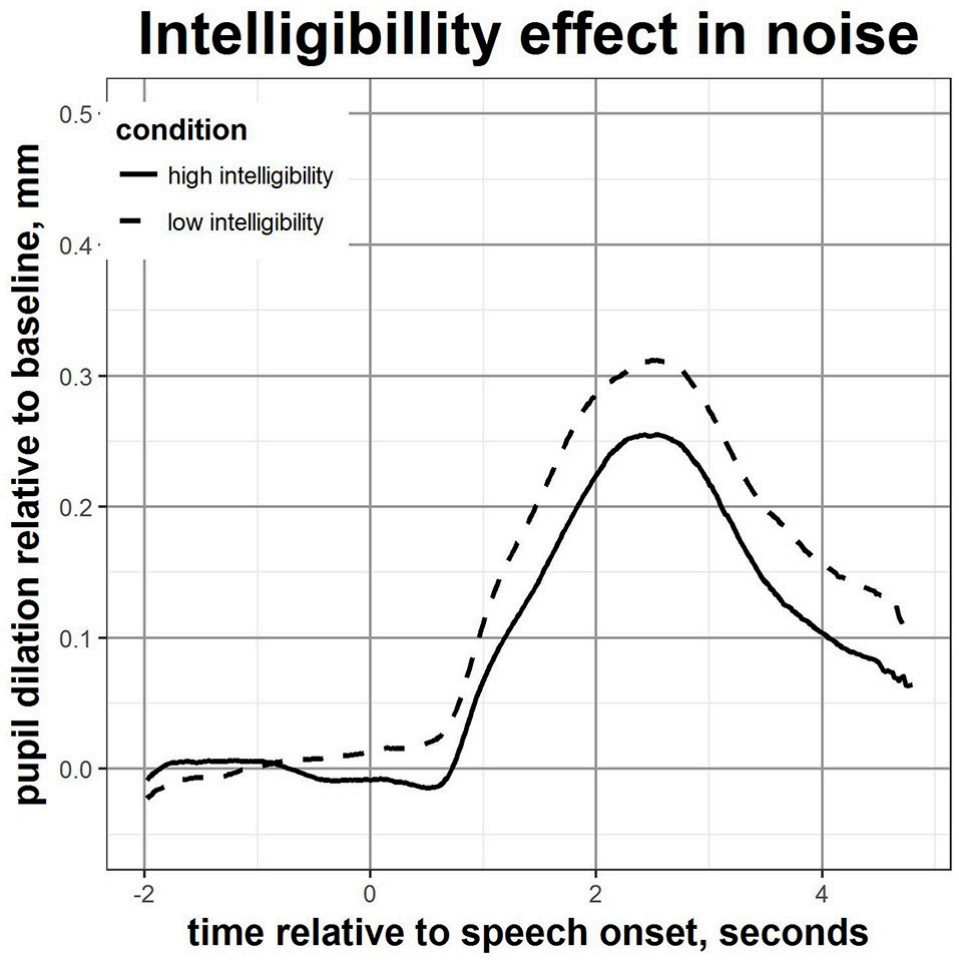
Mean pupil response over time during speech perception in noise for all participants in high and low intelligibility condition.

2x2 mixed-design ANOVAs with intelligibility level (high vs. low) as within-subjects factor, and language background (native vs. non-native) as between-subjects factor were used to investigate the effects of language group and intelligibility level on pupil measures. Figure 2 displays the effects of language group on the time-curves of the event-related pupil dilation, for the high and low intelligibility conditions. Figure 3 displays the main effect of intelligibility level (high vs. low) on the mean pupil dilation over time for all participants. The mean pupil dilation was found to differ significantly both across intelligibility levels [*F*_(1, 48)_ = 10.87, *p* = 0.002] and across language group [*F*_(1, 48)_ = 7.60, *p* = 0.008], however the interaction between the two factors was not statistically significant. The mean pupil change in diameter relative to the baseline was greater for non-native compared to native listeners, and greater for the low compared to the high intelligibility condition.

The same pattern of results was also found when analysing the peak pupil dilation over the baseline. A mixed-design ANOVA showed a main effect of intelligibility [*F*_(1, 48)_ = 9.45, *p* = 0.003] and of language group [*F*_(1, 48)_ = 5.18, *p* = 0.027]. The maximum dilation after stimulus presentation was greater for the low compared to high intelligibility conditions, and greater for non-native than for native speakers. However, there was no significant interaction between these factors so changes in pupil dilation across conditions differing in intelligibility levels did not differ as a function of language group. For the baseline, only the main effect of intelligibility level [*F*_(1, 48)_ = 4.30, *p* = 0.043] was significant: the baseline pupil diameter was greater for the low compared to high intelligibility condition. No statistically significant differences in the latency of the peak were observed, both across test condition and language group.

##### Order effect

**Figure 4:**
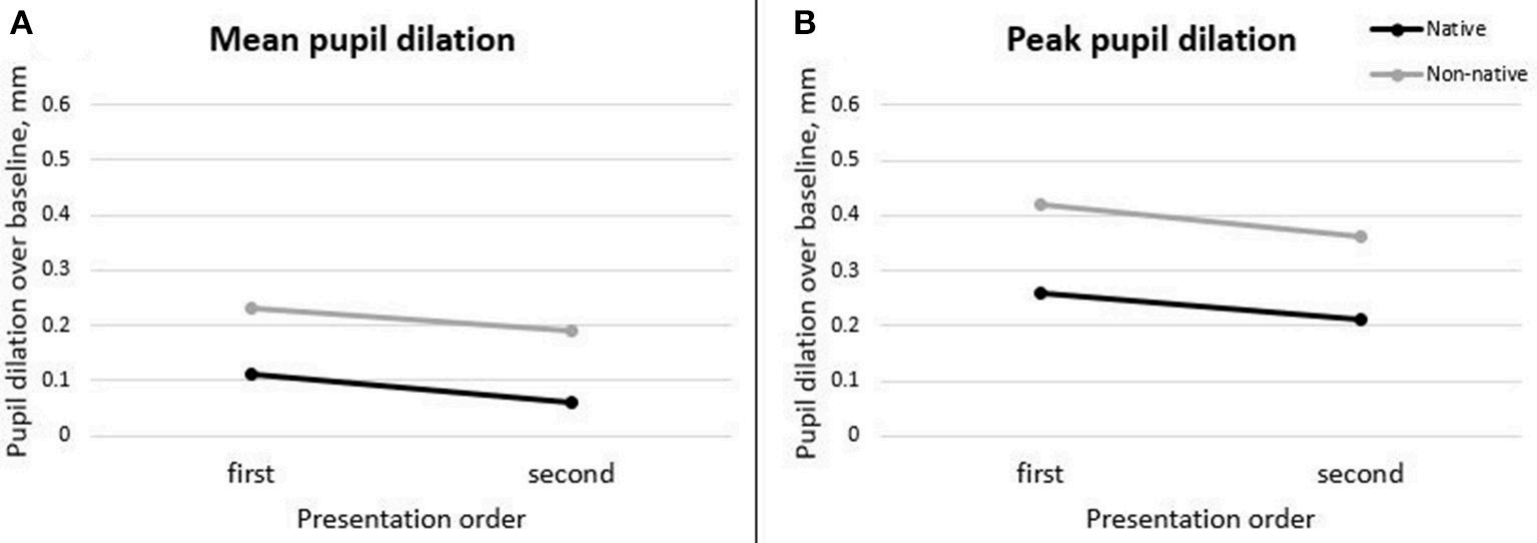
Mean **(A)** and peak **(B)** pupil dilation across test presentation order in noise, for native and non-native listeners.

The effect of order of presentation of the two noise conditions was investigated. To do so, we organised the data according to the presentation order, without taking into account the intelligibility level. 2x2 mixed-design ANOVAs with presentation order (first and second) as within-subjects factor, and language background (native vs. non-native) as between-subjects variable were used to investigate the effects of presentation order on pupil measures for native and non-native listeners. Mean and peak pupil dilation (see Figure 4) differed significantly both across presentation order [*F*_(1, 48)_ = 9.88, *p* = 0.003 and *F*_(1, 48)_ = 10.72, *p* = 0.002 respectively] and across language group [*F*_(1, 48)_ = 7.60, *p* = 0.008 and *F*_(1, 48)_ = 5.18, *p* = 0.027], as already reported in the previous section. The mean and peak pupil change in diameter relative to the baseline were greater for non-native compared to native listeners, and greater for the first compared to the second session in noise. The interaction between the two factors was not found to be statistically significant. No statistically significant differences in the latency of the peak were observed, both across order of presentation and listener type. Order effect was also investigated on the baseline pupil diameter. No main effect of language background and presentation order was found. However, the interaction between presentation order and language group was marginally significant, *F*_(1, 48)_ = 3.90 and *p* = 0.054. This suggests that the order of presentation is likely to have had a different effect on the baseline pupil measure in native and non-native listeners. Follow-up Bonferroni-adjusted pairwise comparisons indicated that only for native listeners the pupil baseline dilation was significantly greater during the second compared to the first test in noise presented (*p* = 0.047), while there was no significant effect of presentation order for non-native listeners. Nevertheless, this was not confirmed by the additional analyses performed using mixed-effect modelling (see Appendix in Supplementary Material).

#### Individual differences effect

Individual differences in intelligibility scores and pupil response were investigated both for all participants and for non-native listeners only. A series of stepwise regression analyses were performed in order to investigate whether the background measures that had been collected were correlated with listeners' performance and listening effort. In this section we report the obtained results, however it is important to note that none of the predictors considered explained a high percentage of the variance observed in speech perception performance and pupil response. Moreover, a lack of consistency in predictors was found across dependent variables. Lastly, additional multilevel modelling analyses performed on the data (see Appendix in Supplementary Material) did not reveal any significant effect of individual differences on the pupil measurements. For these reasons, we believe the following results should be interpreted with caution. We focused the regression analyses on the conditions in noise, since the performance was equated for intelligibility across language groups for these conditions. Moreover, as previously discussed, the relationship between individual cognitive abilities and speech perception processing is more likely to be stronger in more challenging listening conditions.

We first analysed the impact of cognitive abilities on behavioural results and pupil response across all listeners. Six individual stepwise regression analyses were run, considering each of the two noise conditions and each of the following dependent variables: SNR level, peak pupil dilation, mean pupil dilation. In each of those regressions, the scores for forward and backward digit span and for the short term phonological memory test were entered as independent variables. Results showed that for the low intelligibility condition, the final model for estimated SNR and pupil peak dilation included the performance on the forward digit span only, with *R*^2^ = 0.138 and *R*^2^ = 0.81 respectively. A better performance on the forward digit span test resulted in a lower SNR (i.e., better performance) and in a smaller peak pupil dilation. However, for the high intelligibility condition, the final model included short term phonological test results which predicted SNR (*R*^2^ = 0.160): a greater phonological memory capacity was linked to a lower SNR. Second, analyses were re-run for the data from non-native listeners only. The following individual characteristics were entered as independent variables: accent rating, length of residence, overall English use, self-reported English knowledge, digit span forward and backward and short term phonological memory test results. When non-native listeners only were considered, results showed that only the score obtained for the accent rating significantly contributed to predict the estimated SNR level for the low intelligibility test condition (*R*^2^ = 0.168): an accent perceived as more foreign predicted a greater SNR level, i.e., worse performance. Lastly, the performance on the backward memory span was the only significant predictor of the mean pupil dilation during the high intelligibility speech perception test (*R*^2^ = 0.167): a better performance on the backward digit span test predicted a greater mean pupil dilation. The variance of inflation factor was smaller than 2 for each regression coefficient considered, therefore we can assume that the regression results presented were not affected by multicollinearity. Table 7 shows the results for all the significant predictors reported above.

**Table 7:** Effect of individual differences on the behavioural and pupil results in noise, stepwise regression results.

**Dependent variable**	**Predictor**	***R*^2^**	**B**	**Std. error**	**Std. beta**	***F***	***t***	**Sig**.
SNR high int. all listeners	Short-term phonological memory	0.160	−0.537	0.178	−0.400	9.155	−3.026	0.004
SNR low int. all listeners	Forward digit span	0.138	−1.008	0.363	−0.372	7.702	−2.775	0.008
Peak pupil low int. all listeners	Forward digit span	0.081	−0.057	0.028	−0.285	4.250	−2.062	0.045
SNR low int. non-native	Accent rating	0.168	1.204	0.535	0.410	5.062	2.250	0.034
Mean pupil high int. non-native	Backward digit span	0.167	0.063	0.028	0.409	5.025	2.242	0.034

## Discussion

This study assessed the effect of speech intelligibility levels and language background on listening effort, as measured by means of pupil response. The main findings of the experiment are:

1. Pupil response is greater for non-native compared to native listeners during speech perception in quiet, and in noise when intelligibility levels for the two groups of listeners are matched.
2. Pupil response is not differentially modulated by intelligibility level for native and non-native listeners.
3. Pupil response is greater for low compared to high intelligibility levels.
4. The order of test presentation modulates pupil response in native and non-native listeners.

The first and third findings are in line with predictions, while the second is not.

As hypothesised, pupil response (mean and peak dilation relative to baseline) was greater for non-native compared to native participants. This is in line with previous research in the field of second language perception (Schmidtke, 2014); and it also expands the limited literature about second language perception using pupillometry, by directly addressing the challenge of non-native sentence perception in adverse listening conditions.

These results confirmed the combined impact of impoverished L2 phonetic discrimination, increased neighbourhood density, and less efficient use of higher level linguistic information on listening effort for non-native listeners, as discussed in detail in the introduction.

We argue therefore that the overall increased listening effort reflected in the greater pupil response for non-native compared to native listeners might be a result of an increased difficulty arising at multiple levels. First, at a perceptual level because of the less accurate phonetic-perceptual discrimination. Second, at a lexical level due to the increased word competition deriving from L1 words activation, and third because of a generally lower L2 linguistic proficiency. These three levels of difficulty not only play an individual role in enhancing the listening effort required to understand a second language, but they also interact with each other. On the one hand, a less accurate perceptual discrimination is detrimental for a fruitful L2 words activation and context exploitation. On the other hand, reduced linguistic proficiency does not allow for an efficient “gap filling” when perceptual information is not accurate enough, or in case of a degraded audio-signal. Therefore, in order to achieve a performance level similar to native listeners, non-native individuals need to rely more heavily on working memory capacity, which results in more effortful listening. One additional factor that might have contributed to the differences in listening effort between the two listeners' groups is the observed difference in the cognitive abilities, as shown by the cognitive tests results.

As predicted, the listening effort reflected by the mean pupil dilation and by the peak dilation relative to the baseline was higher for the low compared to high intelligibility condition. This result is in line with previous research in native listening, also using individual speech reception thresholds, showing that the pupil response during listening to sentences systematically varied as a function of speech intelligibility if extremely low intelligibility levels are excluded (Zekveld et al., 2010; Zekveld and Kramer, 2014). The growing body of evidence in this direction corroborates the idea that speech perception in difficult listening condition is more heavily reliant on the explicit and effortful exploitation of cognitive resources, particularly working memory. Together with our first finding, an increased pupil response for low compared to high intelligibility conditions, also supports the predictions made by the ELU model (Rönnberg et al., 2013).

Contrary to our predictions, pupil response was not differentially modulated in the two different listeners' groups across intelligibility conditions. That is, the additional amount of listening effort required to non-native compared to native individuals was not greater for lower intelligibility levels relative to higher levels. This result might change if a wider range of intelligibility levels is considered. Along the same lines, previous research also did not report a differential effect of noise for native and non-native listeners on the number of simultaneously activated candidate words during speech perception (Scharenborg et al., 2017). Other individual factors more subtle than the mere linguistic background in terms of native vs. non-native might also contribute to modulate the relationship between intelligibility level and listening effort, as suggested by previous pupillometry research. For example, the ability to read partially masked speech has been regarded as being the visual analogue to speech reception threshold in a previous study, and was found to play a role in the modulation of pupil response together with the tendency to give up listening in particularly challenging conditions (Zekveld and Kramer, 2014).

Interestingly, an effect of presentation order across the two tests in noise was found, with a mean and peak pupil dilation higher in the first compared to the following sessions. This is in line with findings from previous research (Zekveld et al., 2010). Moreover, for native listeners only, an order effect was also found to occur for pupil baseline, showing an inverted trend of change: baseline pupil diameter was at its minimum in first test in noise presented and increased in the second session. However, it is noteworthy that this effect was not confirmed by the additional analyses of the data using mixed-effect modelling (see Appendix in Supplementary Material).

Additionally, the effect of individual differences on the behavioural performance and pupil response was explored, both using stepwise regression analyses, and mixed-effects modelling. Overall, results from stepwise regressions showed that when all listeners were considered, a better performance on the memory tests correlated with better speech perception test in noise. Again, this result seems to reasonably support memory involvement during speech perception in noise, since a greater memory capacity would allow a more efficient and less effortful conflict resolution in case of mismatch between the audio stream and the mental word representation. However, when only non-native listeners were included in the analyses, results were less consistent. When considering the results of the regression analyses, a more heavily accented speech production was linked with a worse speech perception ability. Additionally, contrary to what has been found across all listeners, better memory performance was linked with greater mean pupil dilation in the high but not the low intelligibility condition. However, none of these results were confirmed when data were explored by means of multilevel modelling (see Appendix in Supplementary Material).

As previously mentioned, there was a lack of consistency in the regression results across performance levels and listener groups, and between the regression and multilevel modelling results. Given this, and also due to the relatively low degrees of variance explained by the predictors, we believe that it is not appropriate to draw strong conclusions from these individual differences analyses. Moreover, because of the broad recruitment criteria in terms of English proficiency for non-native participants, it was difficult to draw robust conclusions about individual differences impacting on listening effort for L2 listeners. Indeed, objective measures of English proficiency were not available, and it was not possible to divide participants in balanced groups based on proficiency or length of stay criteria. In addition, the working memory measure collected (forward and backward digit span) was not sensitive enough to show great individual variability in a population of healthy participants, so a potential correlation between cognitive abilities and listening effort is difficult to establish based on the available data. Lastly, the accent rating entered in our analyses was based on a single sentence. Although the sentence considered was the same for all participants, and all non-native listeners shared the same L1 background, this might have been not sufficient for an accurate judgement of the degree of the listener's foreign accent. Further studies should address those limitations, using a more careful selection of proficiency and cognitive measures. Additionally, it could be interesting to evaluate differences between native and non-native pupil response at an intermediate level of understanding (e.g., 50 or 60% of intelligibility). Indeed, the maximum peak pupil dilation has been observed at around 50% correct sentence recognition performance (Ohlenforst et al., 2017), signalling that this might be the intelligibility threshold where listeners engage the most with the speech perception task, and where the maximum amount of resources are actively employed.

In conclusion, this study corroborates pupillometry as a sensitive investigation technique to uncover listening effort differences both within and between participants. This measure was sensitive to differences in intelligibility levels and different listener types; this gives the possibility to quantify differences in listening effort even when listener groups are performing at near-ceiling level, as was the case in the quiet condition. Importantly, the present study showed a greater pupil response in non-native compared to native participants, proving that a greater listening effort is required when trying to understand speech in noise even when intelligibility levels are matched. This was the case for proficient non-native listeners who were achieving around 90% intelligibility for speech comprehension in quiet. Therefore, maintaining a good level of performance when understanding speech in noise comes at a much higher cost for non-native listeners. This is likely to have considerable subsequent effect on the ability to perform more than one task simultaneously and to efficiently and quickly recall information in typical communicative environments. As documented for individuals suffering from hearing loss (McGarrigle et al., 2014), it is reasonable to speculate that a prolonged increase in the listening effort needed to attend speech will result in a greater mental fatigue also for all non-native listeners.

Implications of the study are crucial given the constantly increasing number of people living, working and socialising in a country where their second language is spoken. Further research could also help to clarify second language perception mechanisms, allowing a better development of strategies to facilitate both learning in a second language, and the acquisition of a second language itself. As an example, further research could focus on understanding how the speech signal can be artificially enhanced with additional acoustic or contextual information, in order to make it less effortful to process for native and non-native individuals, by minimising the cognitive load. Potential applications include the possibility of improving PA systems or telecommunications, making important messages easier to understand for everyone even under stressful circumstances or under cognitive load (e.g., in case of emergency or in the workplace). As a step further, the advantage gained by the utilisation of different enrichment approaches in terms of reduced listening effort could be evaluated and targeted for various groups of individuals with different specific needs, ranging from children, adults with hearing impairments and second language learners.

## Author contributions

GB was primarily responsible for the concept and design of the study and VH made contributions to both these elements. GB collected and analysed the data and both authors contributed to the interpretation of results. GB wrote the first draft of the manuscript and VH critically revised its final version. Both authors read and approved the submitted version and agree to be accountable for all aspects of the work.

### Conflict of interest statement

The authors declare that the research was conducted in the absence of any commercial or financial relationships that could be construed as a potential conflict of interest.
