# Supplementary material for: Listening Effort During Sentence Processing Is Increased for Non-native Listeners: A Pupillometry Study
Source: Front Neurosci. 2018 Mar 13;12:152. doi: 10.3389/fnins.2018.00152 (PMC5859302; doi:10.3389/fnins.2018.00152)
Supplement: Supplementary file 1 [file DataSheet1.docx]

# APPENDIX

In this appendix we present the results obtained using mixed-effect modelling to investigate the effects of individual factors on the pupil measures during the condition in noise. Those analyses were performed using the lme4 package in the R environment (Bates, Mächler, Bolker, & Walker, 2014; R Core Team, 2017).

For each dependent variable (mean and peak pupil dilation, peak latency, and baseline), we began with a saturated model that included interaction terms for all as fixed effects with random intercepts and slopes. Due to non-convergence, we simplified the models hierarchically from most complex to least complex, followed by forward entry of random slopes for the fixed effects that were retained in the initial backward elimination. The resulting converged models for all four variables included the following fixed effects: intelligibility level (2: high and low), language background (2: native and non-native), presentation order (2: first and second), forward digit span, backward digit span and short term phonological memory test. Participant was included as random effect but no random slopes. The maximal model only included up to two-way interactions between intelligibility level and language background/forward and backward digit span/phonological memory and between language background and presentation order/forward and backward digit span/phonological memory.
We compared model residuals via chi-square tests (α = .05) from the most complex models (containing the largest interaction term) to the least complex models (containing only single terms). If an interaction term was significant, we included all lower level effects involved in the interaction in the final model.

Results for the dependent variables considered were as follows: 
Mean: the final model included fixed effects of intelligibility level (β= 0.05, SE=0.01, t=3.59, p<.001), language background (β= 0.13, SE=0.06, t=2.76, p=.006), and order of presentation (β= 0.05, SE=0.01, t=3.39, p=.001). This replicates the results obtained from the ANOVAs analyses, showing that overall the mean pupil dilation was greater for the difficult compared to the easy intelligibility condition, for non-native compared to native listeners, and for the first compared to the second session in noise.
Peak: the final model included fixed effects of intelligibility level (β= 0.05, SE=0.02, t=3.34, p=.001) and order of presentation (β= 0.05, SE=0.02, t=3.50, p<.001). Language did not reach significance (p=.071). Overall, the peak pupil dilation was greater for the difficult compared to the easy intelligibility condition and for the first compared to the second session in noise. This is in line with the results of the ANOVA, with the exception that in the previous analyses also language had a main effect on the peak pupil dilation. 
Latency: none of the terms were found to be significant, in line with our previous analyses. 
Baseline: the final model only included fixed effects of intelligibility level (β= 0.07, SE=0.07, t=2.17, p=.030). The baseline pupil diameter was greater for the low compared to high intelligibility condition. In the previous analyses, in addition to a main effect of intelligibility level, we also found a marginally significant interaction between language background and presentation order that is not confirmed here.
None of the three memory measures available for all participants (forward and backward digit span and short term phonological memory test) improved the fit of the model for any of the dependent variables.

Additionally, in order to investigate the effect of accent rating, length of residence, overall English use and self-reported English knowledge on the pupil measure, we ran the analyses with non-native listeners only for all four dependent variables (mean, peak, latency of the peak and baseline).
The resulting converged models for all four variables included the following fixed effects: intelligibility level (2: high and low), accent rating, length of residence, English use and self-reported English knowledge. Participant was included as random effect but no random slopes. The maximal model only included up to two-way interactions between condition and background measures.

Results confirmed a fixed effects of intelligibility level on the mean, peak and latency of the peak (β= 0.06, SE=0.02, t=3.43, p=.001; β= 0.06, SE=0.02, t=2.95, p=.003 and β= -1.50, SE=0.75, t=-2.01, p=.040, respectively for the three variables). When considering the pupil baseline, the final model included a significant interaction between intelligibility level and self-reported English knowledge (β= 0.09, SE=0.03, t=2.63, p=.009) together which the fixed effects of intelligibility level and self-reported English knowledge which did not reach significance. However, follow-up regressions did not reveal any significant effect of the level of self-reported English knowledge on the pupil baseline diameter, either for the easy or the difficult intelligibility condition.
